# Connecting Text Classification with Image Classification: A New Preprocessing Method for Implicit Sentiment Text Classification

**DOI:** 10.3390/s22051899

**Published:** 2022-02-28

**Authors:** Meikang Chen, Kurban Ubul, Xuebin Xu, Alimjan Aysa, Mahpirat Muhammat

**Affiliations:** 1 College of Information Science and Engineering, Xinjiang University, Urumqi 830046, China; xj_ckk@stu.xju.edu.cn (M.C.); xuxuebin@xju.edu.cn (X.X.); 2 The Key Laboratory of Multilingual Information Technology, Xinjiang University, Urumqi 830046, China; alim@xju.edu.cn; 3 International Cultural Exchange College Xinjiang University, Xinjiang University, Urumqi 830046, China; xmahpu@xju.edu.cn

**Keywords:** natural language processing, implicit sentiment analysis, text classification, image classification, data preprocessing

## Abstract

As a research hotspot in the field of natural language processing (NLP), sentiment analysis can be roughly divided into explicit sentiment analysis and implicit sentiment analysis. However, due to the lack of obvious emotion words in the implicit sentiment analysis task and because the sentiment polarity contained in implicit sentiment words is not easily accurately identified by existing text-processing methods, the implicit sentiment analysis task is one of the most difficult tasks in sentiment analysis. This paper proposes a new preprocessing method for implicit sentiment text classification; this method is named Text To Picture (TTP) in this paper. TTP highlights the sentiment differences between different sentiment polarities in Chinese implicit sentiment text with the help of deep learning by converting original text data into word frequency maps. The differences between sentiment polarities are used as sentiment clues to improve the performance of the Chinese implicit sentiment text classification task. It does this by transforming the original text data into a word frequency map in order to highlight the differences between the sentiment polarities expressed in the implicit sentiment text. We conducted experimental tests on two common datasets (SMP2019, EWECT), and the results show that the accuracy of our method is significantly improved compared with that of the competitor’s. On the SMP2019 dataset, the accuracy-improvement range was 4.55–7.06%. On the EWECT dataset, the accuracy was improved by 1.81–3.95%. In conclusion, the new preprocessing method for implicit sentiment text classification proposed in this paper can achieve better classification results.

## 1. Introduction

Sentiment analysis is a basic task in natural language processing. Because sentiment analysis can be widely used in specific practical tasks such as comment analysis [1], public opinion analysis [2], mental health analysis [3], recommender systems [4] and spam identification, it has great development prospects. Sentiment analysis tasks are generally divided into explicit sentiment analysis and implicit sentiment analysis. Generally speaking, there are clear sentiment words in explicit sentiment text sentences, which provide obvious sentiment clues for preliminary sentiment analysis. Therefore, it is relatively easy to distinguish the sentiment polarity of these sentences through classical machine learning methods or popular deep learning methods. However, people often express their feelings in an implicit and obscure way in their daily lives. Despite this, implicit sentiment texts, that is, language fragments (sentences, clauses or phrases) that express subjective emotions but do not contain explicit sentiment words, have rarely attracted researchers’ attention. Moreover, the research on implicit sentiment text analysis has also made slow progress. One of the important reasons for this is that the existing text-processing methods cannot extract the implicit sentiment clues contained in the implicit sentiment text. The traditional method based on sentiment words [5] cannot effectively process the text. Another important reason is that in the existing processing methods for implicit sentiment text, the elimination method of redundant words of implicit sentiment needs to be strengthened. According to a survey, the proportion of implicit sentiment in subjective sentences in various fields is as high as 15–20% [6,7]. At present, implicit sentiment analysis has become one of the core problems in the field of natural language processing [5,7].

Please refer to the following examples of explicit and implicit sentiment sentences:

E1: 我们在一起每天都是**开开心心**的。(“We are **happy** every day together.” Note: explicit sentiment sentences; explicit sentiment word: **happy**; sentiment label: **positive.**)

E2: 果然是传说中的奇女子，琴棋书画样样精通啊。(“Sure enough, she is a legendary woman. She is proficient in piano, chess, calligraphy and painting.” Note: implicit sentiment sentence; no sentiment words; sentiment label: **positive.**)

E3: 我的袜子洗了两遍还全是沙。(“My socks have been washed twice and are still full of sand.” Note: implicit sentiment sentence; no sentiment words; sentiment label: **negative.**)

Sentence E1 is an explicit sentiment sentence. The explicit sentiment word “开开心心 (happy)” can be used as an important clue to identify the sentiment polarity of this sentence. Sentence E2 and Sentence E3 are both implicit sentiment sentences. Sentence E2 show a kind of praise by expressing surprise at the talent displayed by the protagonist. Sentence E3 expresses an implicit negative sentiment by describing the fact that the sand in the shoes cannot be cleaned. It can be seen from these examples that explicit sentiment sentences contain clear sentiment words. Therefore, it is easier to judge the sentiment polarity of such sentences through classical machine learning methods or popular deep learning methods. However, in the face of implicit sentiment text, the existing methods based on sentiment words have difficulty obtaining the implicit sentiment polarity clues contained in the sentence.

Considering that it is difficult for implicit sentiment analysis to classify implicit sentiment text based on the explicit sentiment dictionary, a new preprocessing method for implicit sentiment text classification is described in this paper. With the help of a deep learning network, it can extract more implicit sentiment clues from the samples and judge whether two different samples belong to the same implicit sentiment category through some subtle features of the samples. At the same time, it has a new problem surrounding what method to use to extract the implicit sentiment clues in the samples, so as to quickly and accurately judge whether different samples belong to the same implicit sentiment category. In the process of research, we noticed some useful characteristics of Oriented Fast and Rotated Brief (ORB) [8,9] feature points in the field of 3D reconstruction. ORB feature points can judge whether the object in the picture is the same object by extracting similar feature points in different input samples. ORB feature points have a series of advantages, such as scale invariance, local invariance and so on. Additionally, ORB feature points have been widely proved to be effective feature points in the field of 3D reconstruction. Therefore, when designing the corresponding network, this paper refers to the idea of ORB feature point extraction to solve the problem we just mentioned. This paper assumes that implicit sentiment words with different sentiment polarities have different sentiment polarity weight ranges. When multiple texts with the same sentiment polarity are aggregated, due to the aggregation of the sentiment clues contained in multiple implied sentiment words with the same polarity, the weight value belonging to this domain of sentiment polarity can be greatly improved. This difference in weight values caused by the difference between different affective polarities can be regarded as an important feature of implicit sentiment polarity classification in implicit sentiment analysis tasks.

In this paper, we propose a new preprocessing method for implicit sentiment text classification. We named this method Text To Picture (TTP). TTP draws on the idea of ORB extracting high-quality feature points from samples and applies this idea to the field of implicit sentiment classification; that is, after data enhancement processing of the text, the text is processed by pixel-value conversion, image multivaluing and other processing methods, so as to achieve an effect similar to ORB feature point extraction. Specifically, the samples after data enhancement will eliminate invalid pixels through the set frequency threshold in the process of TTP, converting word frequency value into pixel value, so as to obtain an image with obvious implicit sentiment polarity characteristics, and input it as a new sample to the image-processing network. In the process of selecting an image-processing network, considering the difficulty of extracting implicit sentiment clues in implicit sentiment sentences, fine-grained analysis of samples is needed in sentiment analysis. Therefore, this paper selects the relevant networks in the field of facial micro-expression recognition. To summarize, TTP aggregates the sentiment polarity words in the same sentiment polarity text by converting text into pictures and enhances the weight value of the same sentiment polarity in the input sample, so as to better highlight the sentiment polarity difference between different implicit sentiment texts in the input sample.

The main contributions of this paper are as follows:

1. A simple and effective implicit sentiment text data preprocessing method is proposed, which innovatively converts text features into picture features, so as to better extract the differences between different implicit sentiment polarities;
2. A new research direction of implicit sentiment analysis tasks is proposed: The innovative combination of the two fields of text analysis and image analysis.

The rest of this paper is arranged as follows: the second part introduces related work. The third part introduces the working principle of TTP in detail. The fourth part expresses the experiment and its analysis. Finally, the fifth part relays the conclusion of this paper

## 2. Related Works

In this part, we mainly introduce the research of implicit sentiment analysis, Synchronous Localization And Mapping (SLAM) and facial micro-expression recognition.

### 2.1. Implicit Sentiment Analysis

This section briefly describes the development of implicit-sentiment analysis. At the same time, some better processing methods and technologies in the field of implicit-sentiment analysis are introduced.

In the field of natural language processing, in order to encode the text input into the network, early experts and scholars either proposed to use one-hot coding to mark the word vector, proposed to use the word package to represent a piece of text, or proposed to use the Term Frequency Inverse Document Frequency (TF-IDF) algorithm to mine the information in the text before coding. With the deepening of research in the NLP field, Tomas Mikolov et al. [10] proposed a Word2Vector method with higher performance, which promoted the method of word vector processing in the NLP field to a new stage.

In the aspect of association rules, Mankar and Ingle [11] used association-rule mining technology to extract the implicit sentiment features in tourism reviews, constructed the co-occurrence frequency matrix between explicit features and opinion words, and extracted the implicit sentiment features in the dataset based on the co-occurrence frequency matrix. Jiang et al. [12], based on the association-rule mining technology, extended the basic rules extracted from the dataset by using the topic model. Then they removed the redundant part of the extracted feature indicator by using the pruning method. Finally, the method of combining basic rules with new rules was used to extract the implicit sentiment features from the dataset. Ilya et al. [13], based on association-rule mining technology, improved the original rules used to extract implicit sentiment features in English to a certain extent. By replacing the original algorithm mode in rule representation with the algorithm on a sentence syntax tree, the new rules could be better applied to the task of implicit sentiment analysis in Russian. Liu et al. [14] used the method based on association-rule mining to mark each opinion word without explicit features in the dataset as implicit features. They also extracted a set of association rules from the annotated dataset. Finally, they translated the extracted rules into language patterns and used them for implicit feature extraction. Although these technologies are effective, the association rules that these methods need to design are more complex, and the cost of these methods is higher than the performance advantages they can offer.

### 2.2. Research on SLAM

This section briefly introduces the development history of and existing research on SLAM, as well as some technologies involved in SLAM. Then, it points out the role of ORB feature point extraction in SLAM.

SLAM originated in 1986, and most of the early visual SLAM programs were based on filtering methods. After continuous development and research, relevant staff found that SLAM’s problem was the quick solving of a sparse matrix. The mathematical method for solving sparse matrixes greatly reduces the computational complexity of SLAM. With the continuous development of SLAM, Murray and Klein proposed a breakthrough method in visual SLAM, namely PTAM [15] (Parallel Tracking And Mapping). This method is a parallel algorithm that divides attitude tracking and mapping into two threads, which is very suitable for working in a small workspace. ORB-SLAM [16] and its extended version ORB-SLAM2 [17] were proposed by Mur-Artal et al. They divide tasks into three parallel threads: tracking, local mapping and closed-loop detection. Presently, they have become a classic processing flow in the field of SLAM.

In SLAM’s keyframe-based method, only a few selected frames are generally used to estimate the map. The information from the intermediate frame is discarded. This method can perform more costly but more accurate BA (bundle adjustment) optimization on keyframes. The keyframe-based technique is more accurate than the filter-based method at the same computational cost [18]. Based on these ideas, ORB-SLAM [19,20] uses ORB features to solve the problem that the BRIEF descriptor does not have rotation invariance. By using descriptors in ORB to provide short-term and medium-term data association, the construction of common views is realized to limit the complexity of tracking and mapping. This method reduces the amount of calculation in SLAM and increases the accuracy of drawing.

### 2.3. Facial Micro-Expression Recognition

This part mainly introduces the development of facial micro-expression recognition in recent years and briefly describes some expression-recognition technologies.

In the early 21th century, facial micro-expression recognition began to be studied. With the continuous development of machine learning technology, many micro-expression detection and analysis algorithms are emerging. The most extensive micro-expression detection and analysis method first divides the face into several specific regions. Then the methods of feature extraction and existing classifiers are used for these specific areas, so as to further search and extract the specific relationship corresponding to the specific facial micro-expression features. At present, the method of deep learning is not commonly used in the field of facial micro-expression recognition. The main reasons for this are that the training time is too lengthy and a large number of datasets need to be used to train the system. Among several specific areas divided, because the micro-expression (ME) region is characterized by subtle and rapid change, the difficulty of the existing algorithms is in how to locate the onset frame, apex frame and offset frame accurately and quickly. Yante et al. [21] statistically analyzed the change rate of pixel value in the picture in the frequency domain, then used the information obtained in the frequency domain to locate the apex frame and analyzed the facial micro-expressions by using the local feature information and global feature information of facial micro-expression. Puneet [22] analyzed the spatial and temporal features of faces and used feature coding to encode subtle facial features and then input them to 2D convolutional neural network for recognition. Through the combination of feature coding and convolutional neural network Puneet achieved better ME recognition performance. Li et al. [23] proposed a no-training method based on feature difference contrast and peak detection to identify ME regions. On the other hand, Ma et al. [24] further improved the performance of start frame positioning by using the histogram of directional optical flow.

In order to improve the performance of ME region recognition and analysis, the existing facial micro-expression recognition algorithms have increasingly tended to focus on pixel-level feature point extraction. At present, these pixel-level feature point extraction methods have achieved good results in analyzing subtle facial expressions.

## 3. Method

In implicit sentiment analysis tasks, because there is no explicit emotional word as the clue for implicit sentiment analysis, the traditional dictionary-based method is invalid. We must use other methods to analyze and process the implicit sentiment text. After extensive research, this paper proposes a novel and effective implicit sentiment text preprocessing method. The idea of this method comes from the method of ORB feature point extraction in SLAM. Through the extraction of sentiment features from the same implicit sentiment polarity text, we can increase the differences between different sentiment polarities.

**Figure 1:**
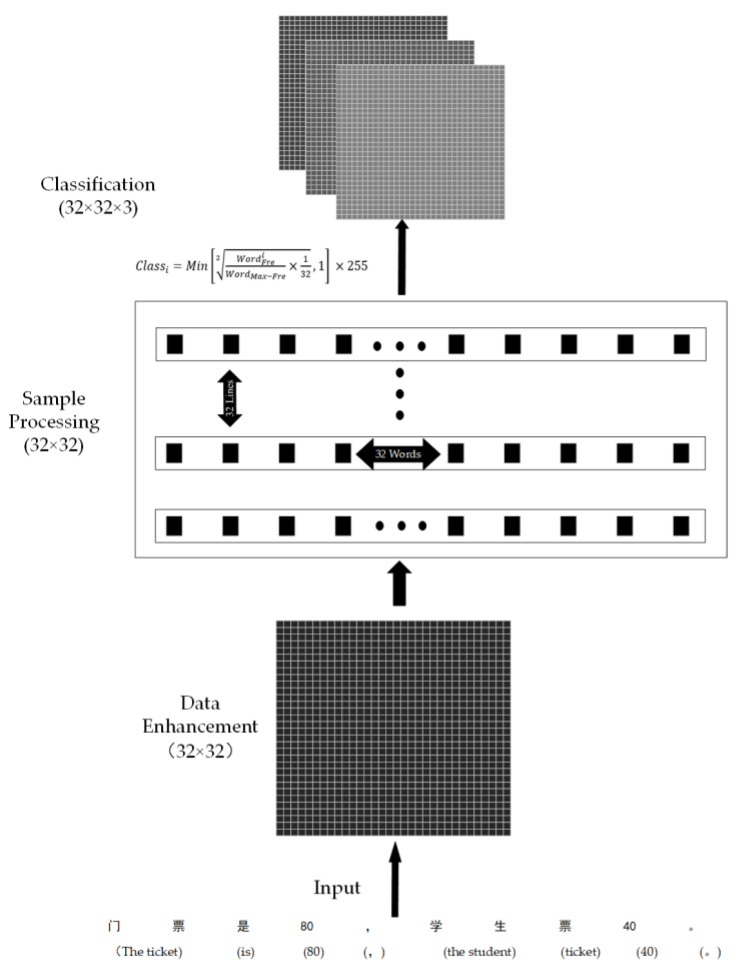
TTP flow diagram.

The implicit sentiment text preprocessing method proposed in this paper consists of three parts, as shown in Figure 1.

- **Data Enhancement:** Data enhancement is aimed at the problem that there are few sentiment polarity clues contained in a single implicit sentiment text and there are no obvious differences in the weight of sentiment polarity between different implicit sentiment texts. Before extracting sentiment polarity cues from the input implicit sentiment text, TTP enhances the text data of the input samples, and the number of samples after data enhancement is 32 times the original. By aggregating 32 samples after data enhancement to a certain extent, this paper highlights the differences of total weight between different sentiment polarities. The data enhancement method used in this paper is the Natural Language Processing Chinese Data Augmentation (NLPCDA) module [25]. The NLPCDA module supports ten different data enhancement methods. The six data enhancement methods used in this paper are as follows:
  1. Random entity replacement;
  2. Random synonym substitution;
  3. Substitution of random near-synonyms and near-syllable characters;
  4. Random character deletion;
  5. Random permutation of the nearest neighbor characters;
  6. Equivalent substitution of Chinese characters.

NLPCDA module parameter setting: create_num = 5, change_rate = 0.38.

It should be noted that the implicit sentiment polarity in the original sentence will not be changed during data enhancement.

**Figure 2:**
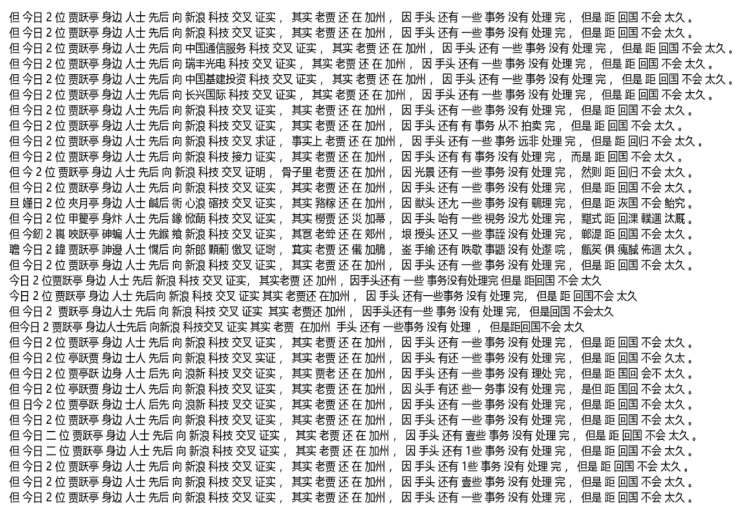
Effect diagram of sample after data enhancement. (Example in the figure: Today, two people around Jia Yueting successively confirmed to Sina technology that Jia Yueting is still in California because there are still some things to deal with, but it will not be long before he returns home.).

In order to better show and explain the effect of input samples after data enhancement, this paper takes Figure 2 as an example for corresponding explanation. The implicit sentiment text shown in Figure 2 was randomly selected from the SMP2019 dataset. From Figure 2, it can clearly be seen that through data enhancement, one sample becomes 32 samples. It is worth noting that Figure 2 illustrates that some samples will be significantly shorter after data enhancement. This situation is caused by the “random character deletion” method mentioned above. The advantage of this is the prevention of overfitting. As for the other five methods used in the process of data enhancement, they do not cause significant changes in sample length.

- **Sample processing:** In this paper, different data enhancement methods are used to enhance the sample data input into the network in turn. Finally, each sample will be enhanced to 32 times the original data. While enriching the sentiment cues in implicit sentiment texts, it is inevitable to introduce some words that have nothing to do with implicit sentiment cues. The appearance of these irrelevant words, on the one hand, increases the amount of calculation of the network and, on the other hand, affects the discrimination accuracy of the subsequent network to the sentiment polarity of the sample. To solve this problem, this paper takes the first 32 words of each sample sentence after data enhancement (if the number of words in the sample is less than 32, add zero; if it is greater than 32, intercept the first 32 words). The advantage of this is that the amount of training data is reduced while retaining the sentiment polarity clues in the implicit sentiment text. Then word-frequency statistics are carried out on the samples in the training set to add two words to word-frequency mapping dictionaries; one of the mapping dictionaries comes from the training set before data enhancement and the other from the training set after data enhancement. In the end, the redundant values of the two-word frequency dictionaries are eliminated (“redundant values” refers to meaningless symbols and their corresponding frequency values in the word frequency dictionary). In this part, a 32 × 32-dimensional character matrix and two-word frequency mapping dictionaries are obtained.
- **Classification:** In order to better highlight the sentiment polarity clues contained in the samples, this paper proposes and uses a novel classification function to classify different categories of words. At the same time, in order to make the classified frequency matrix better processed by the subsequent network, we converted it into an image for output.

The specific steps are as follows:

1. The first step is to classify the words in the statistical dictionary. The basis for classifying words is shown in Formula (1).

(1) $$Class_{i}=Min\left[\sqrt[{2}]{\frac{{Word}_{Fre}^{i}}{{Word}_{Max-Fre}}\times \frac{1}{\mathrm{32}}}\;\;,\;1\;\right]\times 255$$

where $Word_{Fre}^{i}$ represents the frequency value of the word currently processed. This value comes from the statistical value after data enhancement. $Word_{Max-Fre}$ represents the maximum frequency value in the dictionary after deleting the frequency value of punctuation in the statistical dictionary. This value comes from the statistics before the data enhancement. All statistics are from the training set. $Class_{i}$ is the calculated result after rounding the i-th word. Its maximum value is 255 and its minimum value is 0. Min[] means taking the minimum value in parentheses. The $\frac{1}{32}$ in the $\sqrt[{2}]{\;\;\;}$ is to balance the impact of data enhancement on word frequency statistics. The $\sqrt[{2}]{\;\;\;}$ is to eliminate the category difference between different words caused by too many words and the interference of words with frequencies that are too small on implicit sentiment polarity cues. After using the above function for classification, a word classification table can be obtained.

In order to better explain Formula (1), an example is as follows: Suppose that the statistical value (Unit: Times) of the word “猫 (cat)” in the original training set is A. After data enhancement of the training set i times, we count the number of times the word “猫 (cat)” appears in the new training set again. Assuming that the statistical value becomes B at this time, we can obtain the following:

(2) $$Class_{cat}=Min\left[\sqrt[{2}]{\frac{B}{A}\times \frac{1}{i}}\;\;,\;1\;\right]\times 255$$

**Figure 3:**
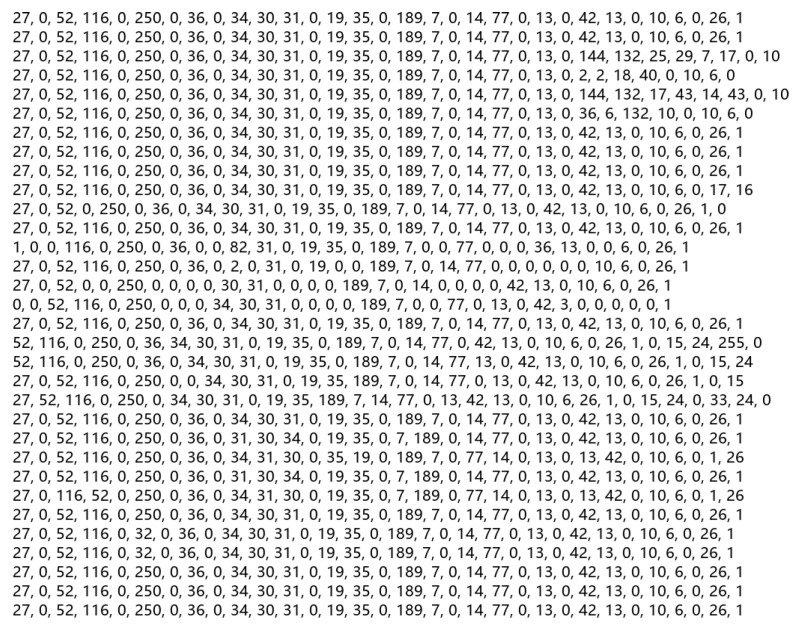
Schematic diagram of numerical matrix.

**Figure 4:**
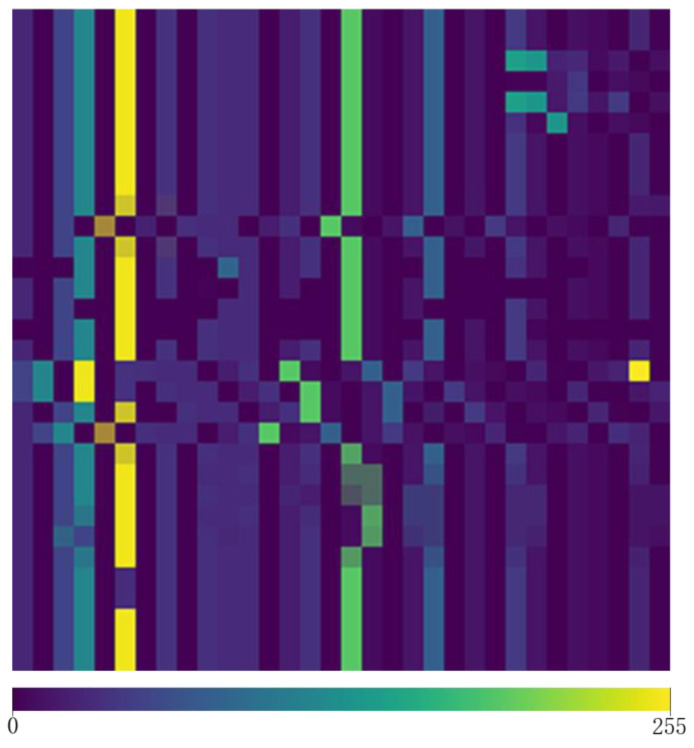
Final effect diagram after processing input samples.

2. In the second step, according to the word classification table obtained in the first step, replace the characters in the character matrix with the corresponding classification value and then obtain a 32 × 32-dimensional numerical matrix. Figure 3 shows the effect diagram of the numerical matrix obtained in this step. It should be noted that each number in Figure 3 corresponds to the words in the same position in Figure 2 one by one.
3. In the third step, the numerical matrix obtained in the second step is converted into an image with an aspect ratio of 32 × 32. Figure 4 is the final effect diagram after extracting the implicit sentiment clues in the implicit sentiment text through the operation of the TTP method. The pixel values on each pixel block in Figure 4 correspond to the values at the same position in Figure 3 one by one.

## 4. Experiment and Analysis

In this part, we first briefly introduce the experimental configuration, evaluation indicators and datasets, and then we introduce the relevant experimental results and analysis in detail.

### 4.1. Dataset Introduction

This paper uses the following two datasets to test and validate our proposed method:

- **SMP2019 Dataset:** The SMP2019 dataset is an evaluation dataset that was used in the Chinese implicit sentiment analysis tasks of the SMP2019 Conference (one of the top social media-processing conferences in China) [26]. The data in the dataset were mainly obtained from four websites: (a) The first data source website is currently China’s largest social media and comment platform, Weibo. The data content obtained from Weibo focuses on implicit sentiment, and involves different fields, such as “China Olympic Games”, “China Spring Festival Gala” and “LETV Company”. (b) The second data source is Mafengwo, which is one of China’s famous tourism review platforms. (c) The third data source is another famous tourism review platform in China, Ctrip. (d) The fourth data source is AutoHome (AutoHome is one of China’s famous auto review platforms). The comments extracted from the forum mainly focus on the characteristics of the product and the follow-up services of the product. These data are much longer than the Weibo data, and can obtain more implicit sentiment information of the comment target [27]. Table 1 shows the number of texts of different sentiment categories in the training set, verification set and test set of the SMP2019 dataset used in the experiment.

- **EWECT Dataset:** The EWECT dataset used in this paper is a general microblog dataset. The data in the general microblog dataset are randomly collected in the microblog; the data includes various topics, covering a wide range. The original data comes from Sina Weibo and is provided by Micro-Hotspot Big Data Research Institute. Table 2 shows the number of texts in different sentiment categories in the training set, verification set and test set of the EWECT dataset used in this paper.

### 4.2. Experimental Configuration and Metrics

The equipment used in this experiment included a notebook computer. The processor was Intel Core i7-9750H. All of the reported results were obtained by running the Python code on an NVIDIA GeForce RTX 2060 GPU.

The evaluation index used in this paper is accuracy. The calculation formula is shown in Formula (2). TN (True Negative) is the number of negative classes predicted as negative classes. FP (False Positive) is the number of negative classes predicted as positive classes. FN (False Negative) is the number of positive classes predicted to be negative. TP (True Positive) is the number of positive classes predicted as positive classes. The range of *ACC* is 0–1; the closer its value is to 1, the better the classification effect is.

(3) $$ACC=\frac{TP+TN}{TP+TN+FP+FN}$$

### 4.3. Comparison Experiment

This paper selected seven common models in text classification as the competitive networks. The seven different networks are TextCNN [28], TextRNN [29], TextRNN+Attention [30], TextRCNN [31], FastText [32], DPCNN [33] and Transformer [34]. Considering the classification of samples with different sentiment polarity, this paper used the way of converting implicit sentiment text into pictures. At the same time, the implicit sentiment analysis task needs a fine-grained sentiment feature extraction network. Therefore, this paper selected the facial expression recognition network in the image classification network (this paper used FER-Net [35] as the basic verification network) to analyze the TTP-processed images. The above networks carried out binary-classification implicit sentiment analysis experiments on the SMP2019 implicit sentiment text dataset and EWECT dataset, respectively. Additionally, all the experimental data used this time are the average values calculated after five experiments, so as to ensure the robustness of the experiment. Table 3 shows the experimental results of the SMP2019 implicit sentiment text dataset. Table 4 shows the experimental results of the EWECT implicit sentiment text dataset. In the tables, it can be clearly seen that on the two datasets, the implicit sentiment polarity classification effect obtained by the TTP-processing method proposed in this paper is higher than that of the competitor network. Especially in the SMP2019 dataset, the improvement of classification accuracy of our proposed method is more obvious. Among the seven competitor networks, TextRCNN has the highest accuracy, reaching 81.09%. Nevertheless, the accuracy of our proposed method is still 4.55% higher than it.

The above experimental results show that the TTP method proposed in this paper performs well in the implicit sentiment text binary-classification task. The reasons are as follows: First, due to the complexity of Chinese language expression, it is difficult to enumerate prior knowledge in Chinese implicit sentiment texts by means of manual statistics. Second, we believe that the existing deep learning networks are fully capable of the “autonomous” finding of some semantic links between different kinds of words in a small range after training. The key point surrounds how to transform the semantic expression into a language that can be understood by the machine and how to “compress” thousands of commonly used words into an appropriate “small range”. Third, to solve these two problems, our solution is to map different words into different word categories for coding through Formula (1) proposed in our article. This mapping method does not eliminate the connection between different words in the text. On the contrary, by reducing the number of word types (from thousands to 256), we better highlight the clues with corresponding implicit sentiment in the text. TTP also broadens the processing methods in the field of natural language processing, especially for the task of implicit sentiment text classification, and widens the solution channel of implicit sentiment analysis.

### 4.4. Ablation Experiments

In order to illustrate the effectiveness of the method proposed in this paper, we tested it on the SMP2019 dataset and EWECT dataset, respectively. Table 5 shows the results of ablation experiments on the SMP2019 dataset, and Table 6 shows the results of ablation experiments on the EWECT dataset. In these two tables, ES+FER-Net represents that the text after data expansion is directly converted into picture form and input into the network. This paper takes this as the comparative sample in the ablation experiment. TTP+FER-Net means inputting the pictures of implicit sentiment text after TTP processing into the network. The table shows that the classification accuracy of ES+FER-Net on the two datasets is only about 50%. In sharp contrast, the accuracy of the TTP+FER-Net method is as high as 85.64%. By comparing the classification effect of implicit sentiment, we can know that the way of directly converting the text after data expansion into picture form cannot highlight the implicit sentiment clues in the implicit sentiment text, but, after the implicit sentiment text is processed by the TTP method proposed in this paper, the effect is obviously better than the operation of directly converting the information into pictures. This experiment proves that the TTP processing method proposed in this paper can achieve good results in the task of implicit sentiment text classification.

## 5. Conclusions and Prospects

In conclusion, this paper proposes a new preprocessing method for implicit sentiment text classification. This method processes implicit sentiment text through three parts: data enhancement, sample processing and image classification, and it innovatively combines the two fields of natural language processing and image processing. Through the TTP method proposed in this paper, the relative differences between different sentiment polarities in implicit sentiment text are amplified, and the implicit text information is transformed into pictures with more prominent sentiment polarities, so as to achieve a better effect of implicit sentiment text analysis.

However, during the experiment, a large training set is usually needed to train the network, so that the machine can pay attention to how the different semantic relationships between different words affect the sentiment polarity contained in the sentence. To solve this problem, this paper makes a certain degree of optimization. On the one hand, TTP reduces the overall number of semantic relations by “compressing” the types of words. Compared with the amount of data required before “compression”, it indirectly enables us to obtain a better result with less data. On the other hand, TTP increases the number of sentiment cues contained in the new samples by converting the output results enhanced by the input sample data into images so that the network can more easily notice the sentiment cues hidden in the text.

At the same time, the paper still has some deficiencies in dealing with the finer-grained implicit sentiment text classification task. We also know that the knowledge tree is a method combined with traditional deep learning methods, which can associate concepts with text statements. Next, while studying the relevant contents of the knowledge tree, we will continue to study how to classify different sentiment polarities in implicit sentiment words more effectively and try to add corresponding sentiment analysis and processing modules into the network to optimize or solve this problem.

## Figures and Tables

**Table 1:** The proportions of the implicit experimental dataset for SMP2019.

Dataset	Subset	Positive	Negative
SMP2019	Training	3749	7425
	Development	469	922
	Testing	463	494

**Table 2:** The proportions of the implicit experimental dataset for EWECT.

Dataset	Subset	Positive	Negative
EWECT	Training	4568	12,767
	Development	391	1017
	Testing	810	854

**Table 3:** Implicit sentiment analysis on SMP2019 dataset. (Two classifications: positive, negative).

Dataset	Model	Accuracy (%)
SMP2019	TextCNN	80.67
	TextRNN	79.00
	TextRNN + Attention	78.58
	TextRCNN	81.09
	FastText	79.10
	DPCNN	80.36
	Transformer	79.21
	TTP + FER-Net	**85.64**

**Table 4:** Implicit sentiment analysis on EWECT dataset. (Two classifications: positive, negative).

Dataset	Model	Accuracy (%)
EWECT	TextCNN	77.36
	TextRNN	77.72
	TextRNN + Attention	76.28
	TextRCNN	78.00
	FastText	75.98
	DPCNN	78.12
	Transformer	77.06
	TTP + FER-Net	**79.93**

**Table 5:** Results of ablation experiments on SMP2019 dataset. (Two classifications: positive, negative).

Dataset	Model	Accuracy (%)
SMP2019	FER-Net	50.57
	TTP + FER-Net	**85.64**

**Table 6:** Results of ablation experiments on EWECT dataset. (Two classifications: positive, negative).

Dataset	Model	Accuracy (%)
EWECT	FER-Net	51.32
	TTP + FER-Net	**79.93**

## Data Availability

We use two Chinese implicit sentiment analysis datasets to evaluate a new implicit sentiment text classification preprocessing method proposed in this paper. The two datasets are the SMP2019 dataset and EWECT dataset. The URLs of these datasets are https://www.biendata.xyz/competition/smpecisa2019/; https://smp2020ewect.github.io/ (both accessed on 12 December 2021).
